# Epidermal growth factor receptor pathway mutation and expression profiles in cervical squamous cell carcinoma: therapeutic implications

**DOI:** 10.1186/s12967-015-0611-0

**Published:** 2015-07-25

**Authors:** Sureewan Bumrungthai, Kavita Munjal, Shirish Nandekar, Kumarasen Cooper, Tipaya Ekalaksananan, Chamsai Pientong, Mark Francis Evans

**Affiliations:** Department of Microbiology, Khon Kaen University, Khon Kaen, 40002 Thailand; Department of Pathology, Sri Aurobindo Institute of Medical Sciences, Indore, Madhya Pradesh 453555 India; Department of Pathology and Laboratory Medicine, Pearlman School of Medicine, University of Pennsylvania, Philadelphia, 19104-4283 USA; HPV & EBV and Carcinogenesis Research Group, Khon Kaen University, Khon Kaen, 40002 Thailand; Department of Pathology and Laboratory Medicine, University of Vermont, Burlington, 05405 VT USA; University of Vermont Cancer Center, Burlington, VT 05405 USA

**Keywords:** Cervical cancer, HPV16, EGFR, PIK3CA, KRAS, PTEN, AKT, mTOR, MAPK, ERK1/2

## Abstract

**Background:**

Cervical squamous cell carcinoma (CSCC) is a major cause of female mortality worldwide. This study has examined epidermal growth factor receptor (EGFR) pathway markers that represent actionable pharmacological targets.

**Methods:**

HPV16 positive CSCCs (n = 105 patients) from Madhya Pradesh, India were screened for *KRAS* and *PIK3CA* mutations by PNA-clamp real-time PCR. Immunohistochemistry (IHC) was performed for EGFR, PIK3CA, PTEN, phospho-AKT, phospho-mTOR and phospho-44/42 MAPK (ERK1/2).

**Results:**

*KRAS* mutations were detected in 0/91 (0%) and *PIK3CA* mutations in 19/95 (20.0%) informative specimens: exon 9, E542 (n = 3) and E545 (n = 15); exon 20, H1047R (n = 1). *PIK3CA* mutation detection was associated with older mean patient age [48.2 vs. 56.6 years (P = 0.007)] and with post-menopausal age: 5/45 (11.1%) patients <50 years vs. 14/50 (28.0%) patients ≥50 years (P = 0.045; OR = 3.11). EGFR expression was present in 60/101 (59.4%) CSCCs and was associated with *PIK3CA* mutation detection (P < 0.05) but not age (P > 0.05). EGFR and phospho-AKT staining showed associations with tumor grade and/or lymph node status (P < 0.05). Significant associations were not found for the other study markers (P > 0.05).

**Conclusion:**

These data show that *PIK3CA* mutation acquisition is related to patient age and EGFR expression. The absence of *KRAS* mutations supports the potential of anti-EGFR therapies for CSCC treatment. The relatively high *PIK3CA* mutation rates indicate that PI3K may be a therapeutic target for a significant subset of CSCC patients. Qualitatively distinct IHC staining profiles for the marker panel were noted patient to patient; however, across patients, consistent linear relationships between up- and downstream pathway markers were not observed. Evaluation of the expression status of potential cancer pathway targets may be of value in addition to molecular profiling for choosing among therapeutic options.

**Electronic supplementary material:**

The online version of this article (doi:10.1186/s12967-015-0611-0) contains supplementary material, which is available to authorized users.

## Background

Cervical cancer is the fourth most common and fatal female malignancy worldwide: each year there are an estimated 528,000 new cases and 266,000 deaths from the disease [1]. Around 85% of deaths occur in low or middle income less developed countries (LDCs). The highest mortality rates are found in African and South Asian countries [1, 2]. India ranks first for the overall number of cervical cancer deaths per year: 72,825, which accounts for more than a quarter of the global burden. With respect to mortality rate (15.2/100,000 deaths), India ranks seventeenth [1, 2].

Cervical cancer is a preventable disease. The introduction of national cervical cytology screening programs among more developed countries (MDCs) has greatly reduced disease incidence and mortality rates [3]. Human papillomaviruses (HPV) are well characterized as the cause of cervical cancer and supplementation of cytology screening with HPV testing has also helped improve the detection of precursor lesions and patient management. HPV vaccination programs have been established in many countries. However, LDCs such as India lack the resources and infrastructure to support comprehensive national cervical cytology screening or HPV vaccination programs. Although initiatives are in place, it will likely be many years before screening and vaccination are widely accessible to Indian women, especially in rural areas where the majority of cervical cancer related deaths occur [4, 5]. Consequently, the disease will remain a social and economic burden in India for some time to come. Additionally, cervical cancer continues to impact a significant subset of women from MDCs; in the USA, 12,900 new cases and 4,100 deaths (2.3 per 100,000) are predicted for 2015 [6]. Therefore there is a considerable need for cervical cancer treatments that are effective worldwide.

The epidermal growth factor receptor (EGFR), a receptor tyrosine kinase (RTK), is a well characterized target for anti-cancer therapies for a variety of cancer types [7, 8]. EGFR stimulation activates a tyrosine kinase domain that controls multiple cell growth and division functions including maturation, proliferation, inhibition of apoptosis, angiogenesis and metastasis via pathways downstream of EGFR signaling. The MAPK/ERK and PI3K/AKT/mTOR pathways are of particular clinical interest [7, 8]. EGFR mediated activation of the mitogen-activated protein kinase/extracellular signal-regulated kinase (MAPK/ERK) signaling cascade (KRAS, BRAF, MEK and ERK1/2) mobilizes transcription factors leading to cell proliferation [9–11]. Mutations to the oncogene *KRAS* result in constitutively active KRAS allowing chronic downstream signaling. EGFR stimulation also activates the PI3K/AKT/mTOR pathway that results in translation factor activation protein synthesis and degradation of the tumor suppressor transcription factor FOXO3; PI3K (phosphatidylinositol-4,5-bisphosphate 3-kinase) is also activated by RAS [9, 12–14]. Mutations to the oncogene *PIK3CA* (a sub unit of *PI3K*) may predict responsiveness to PI3K/AKT/mTOR inhibitors and to anti-EGFR therapies [12–16].

Causes of EGFR upregulated expression include receptor overexpression and activating mutations [7]; however, in cervical cancer EGFR mutations are undetectable [14–19] or uncommon [20]. EGFR expression in cervical cancer may also be activated by HPV16 E5 or E6/E7 proteins [21, 22]. Additionally, HPV16 E6 has been shown to cause receptor hyperactivation under conditions of abundant growth factor availability and to increase the internalization of phosphorylated EGFR even in the absence of growth factors; this results in prolonged receptor activation and MAPK and PI3K stimulation [22].

Pharmacologically, there are two modes of EGFR inhibition: anti-EGFR monoclonal antibodies (e.g., cetuximab, panitumumab), or specific inhibitors of the EGFR tyrosine kinase domain (e.g., erlotinib, lapatinib). Clinical efficacy of anti-EGFR therapies has been shown in clinical trials for lung, colon, pancreas and head and neck tumors [7, 11–15]. Anti-EGFR treatment efficacy is impacted by aberrations to downstream elements in the pathway [7–9, 11–15]. *KRAS* is the most frequently mutated member of the *RAS* gene family and encodes a 21 kDa guanosine 5-triphosphate-(GTP-) binding protein early in the MAPK signaling pathway. The most common *KRAS* mutations occur at codon positions 12 and 13 in exon 2, and less frequently in codons 61, 63, 117, 119, and 146. Wild type KRAS protein expression is normally regulated via EGFR and GAP proteins; however, activating *KRAS* mutations are insensitive to EGFR or GAP inhibition resulting in chronic signaling of cells to grow and divide: activated KRAS results in the phosphorylation and activation of ERK1/2 via the activities of B-RAF and MEK 1/2. Phosphorylated ERK 1/2 activates AP-1 family transcription factors such as jun and fos, which on binding to AP-1 result in the expression of cyclins, cytokines and growth factors promoting cell proliferation [7–9, 11, 12, 23]. Novel ERK inhibitors are in development to facilitate treatment of patients with activating *KRAS or BRAF* mutations [23].

EGFR also stimulates PI3K expression and the PI3K/AKT/mTOR cascade resulting in increased transcription, protein synthesis and proliferation [9, 13–15]. The phosphatidylinositol-4, 5-bisphosphate 3-kinase, catalytic subunit alpha (*PIK3CA*) gene encodes the p110α catalytic subunit protein of PI3K. *PIK3CA* mutations occur in a wide range of tumors. In some tumor types *PIK3CA* mutations are frequently associated with *EGFR* or *KRAS* mutations [24, 25] and with a poorer prognosis [25]. A number of drugs targeting PI3K have been developed and are under investigation for therapeutic utility [18–22]. Phosphatase and tensin homolog (PTEN) is tumor suppressor gene that negatively regulates PI3K signaling by protein phosphatase activity. Loss of expression or PTEN mutation is associated with many tumors [15, 26].

**Fig. 1:**
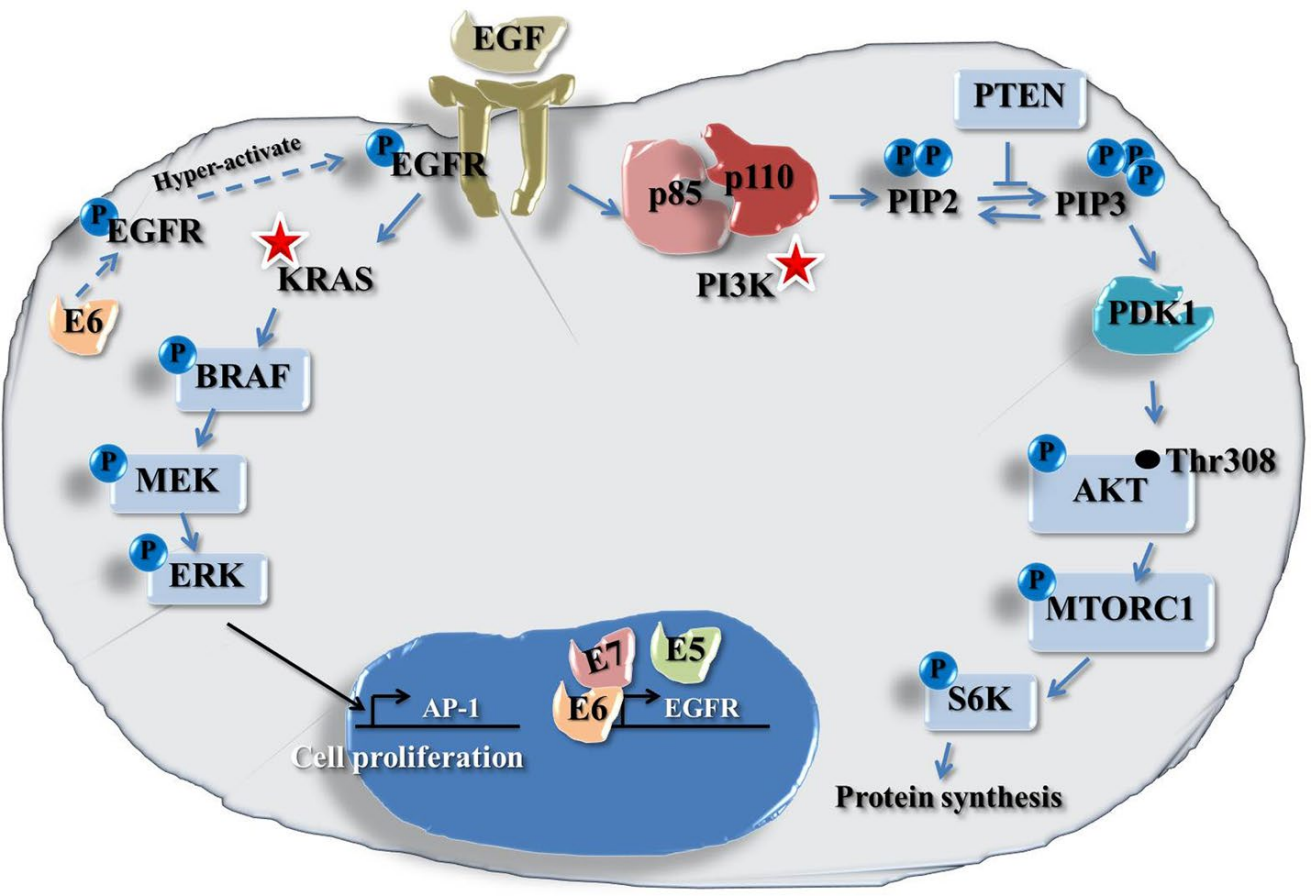
Simplified representation of the EGFR MAPK/ERK and PI3K/AKT/mTOR pathways (see “Background” text for step details).

Active PI3K, phosphorylated through receptor tyrosine kinase or RAS GTP activity, can phosphorylate the 3′ position hydroxyl group of the inositol ring of phosphatidylinositol (PIP2) converting it to PIP3; the serine/threonine protein kinase AKT (Protein Kinase B) binds to PIP3 at the plasma membrane, allowing PDK1 to activate AKT by phosphorylation at Thr308. Phosphorylated AKT is a key regulator of numerous cellular and nuclear process, including the promotion of protein synthesis via a multistep protein cascade that includes activation of mTOR (mechanistic target of rapamycin) and thence activation of the translation factor S6 K that promotes protein synthesis. Deregulators of this pathway include activating mutations in the *PIK3CA* gene (p110 subunit) and inactivation of the phosphatase and tensin homolog (*PTEN*) gene. The *PIK3CA* gene encodes PI3-Kinase that can phosphorylate the 3′ position hydroxyl group of the inositol ring of phosphatidylinositol, a key signal transducer in the PI3K-AKT pathway [12, 13]. An extensive variety of drugs have been developed targeting the PI3K/AKT/mTOR pathway. These include inhibitors of PI3K p110α, AKT, or mTOR, as well as dual PI3K/mTOR inhibitors [12, 13]. A schematic of the EGFR signaling pathway is shown in Fig. 1.

The aims of our study were to examine the biologic expression and potential therapeutic significance of MAPK/ERK and PI3K/AKT/mTOR pathway mediators in Indian cervical squamous cell carcinomas (CSCCs) and to compare these data to similar studies from more developed countries. CSCCs were investigated by PNA-Clamp PCR for *KRAS* and *PIK3CA* mutations and by immunohistochemistry (IHC) for the expression of EGFR, pMAPK (pERK1/2), PIK3CA, PTEN, pAKT, and p-mTOR.

## Methods

### Specimens

The study was conducted with approvals from the Institutional Review Boards of the Sri Aurobindo Institute of Medical Sciences (SAIMS), Indore, Madhya Pradesh, India, and The University of Vermont, Burlington, Vermont, USA; patient informed consent was not required as coded archival specimens from patients already treated to the standard of clinical care were utilized. Formalin-fixed, paraffin-embedded (FFPE) CSCC specimens from 105 patients, untreated other than by surgery, were accrued for the study. All specimens had previously tested HPV16 positive [27]. Patient cohort clinical data is shown in Table 1.

**Table 1:** Characteristics of the analyzed cohort

Clinical characteristics	n = 105
Age range	25–90
Mean age (SD)	49.7 (13.2)
FIGO stage	
Stage I	
T1/T1a1	2
T1b1	65
T1b2	27
Stage II–III	11
Tumor size	
≤2 cm	12
>2–5 cm	77
>5 cm	16
Lymph node status	
Positive	20
Negative	67
No data	18
Histology	
Well	17 (16.2%)
Moderately	74 (70.5%)
Poorly	14 (13.3%)

### DNA extraction

Five to ten 10 µm FFPE tumor sections were cut into 1.5 mL tubes. Microtome blades were cleaned with xylene, alcohol and DNA *AWAY* (Thermo Scientific, Waltham, MA, USA) between specimen blocks. Tissue sections were cleared of paraffin by xylene followed by ethanol washes. DNA was extracted from dehydrated tissues using proteinase K combined with column purification (DNeasy Blood and Tissue kit, Qiagen, Valencia, CA, USA). DNA extracts were quantified and assessed for purity by NanoDrop 2000 spectrophometry (NanoDrop Technlogies, Wilmington, DE, USA). DNA amplification quality was confirmed for all samples by PCR for a 209 base pair β-globin fragment [27].

### Mutational analysis of *PIK3CA* and *KRAS* by peptide nucleic acid (PNA) clamping assay

*KRAS* and *PIK3CA* gene mutation assays were performed by quantitative real-time PCR (SYBR Green) using PNA-Clamp Mutation Detection Kits (PNA Bio Inc., Thousand Oaks, CA, USA): 15-20 ng of purified DNA per sample was assayed using a CFX96 Touch™ Thermal Cycler (Bio-Rad Inc., Hercules, CA, USA). The *KRAS* kit screens for seven common mutations of exon 2 (codon 12: nucleotide changes c.35G>A, c.35G>C, c.35G>T, c.34G>A, c.34G>C, c.34G>T; codon 13: c.38G>A). The *PIK3CA* kit screens for nine common mutations: exon 9 (E542 nucleotide changes: c.1624G>A, c.1625A>G, c.1625A>T; E545: c.1633G>A, c.1634A>G, c.1635G>T) and exon 20 (H1047: c.3139C>T, c.3140A>T, c.3140A>G). Cycling conditions for all assays consisted of pre-denaturation at 94°C for 5 min followed by 40 cycles of denaturation (94°C for 30 s), PNA clamping (70°C for 20 s), annealing (63°C for 30 s), and extension (72°C for 30 s). The PNA-Clamp assay uses DNA primers to amplify across the locus/loci of interest in combination with an internal PNA oligo designed to anneal to the wild type form of the target. Primer extension and amplification is prevented if wild-type *KRAS* or *PIK3CA* is present due to the stability of the PNA/DNA hybrid when sequences perfectly anneal, whereas a single base mismatch is highly destabilizing so permitting primer extension when a mutation is present. Mutation detection assessment was performed according to an algorithm provided with the kit and involves taking into account the cycle threshold (Ct) values of specimen PCRs performed minus a PNA-Clamp oligo compared to the Ct value performed with a PNA-Clamp oligo. Clamping controls of known wild type DNA was performed as a negative control.

### Tissue microarray (TMA) preparation

TMAs were prepared from the 105 CSCC specimens by removing 1.0 mm diameter cores of tumor from the FFPE blocks using a Tissue Arrayer (Beecher Instruments, Inc., Wisconsin, USA). Guided by haematoxylin and eosin (H&E) stained slide-mounted tissues sections ink-marked for tumor areas, blocks were sampled from up to four different regions to control for heterogeneity. All 105 patient samples were contained in a total of five TMA blocks. TMA sections were H&E stained and reviewed by a histopathologist to confirm tumor had been accurately targeted: in two instances, inadequate tumor material was sampled for IHC analyses.

### Immunohistochemistry (IHC)

IHC was performed for six target antigens (Table 2): EGFR, PIK3CA, PTEN, and activated forms of AKT (i.e., phosphorylated at Thr308 by PDK1 that functions downstream of PI3K via conversion of PIP2 to PIP3), mTOR (i.e., phosphorylated at Ser2448 via PI3 kinase/AKT signaling), and MAPK (ERK1/2) (i.e., phosphorylated at Thr202 and Tyr204 by MEK1 and MEK2). The choice of IHC antibody clone was determined by literature and supplier datasheet review and by availability of clones suitable for FFPE applications. In some instances several alternative clone choices were available; it was beyond the resources available for this study to compare alternatives. As regards AKT, complete activation involves phosphorylation at Ser473 and Ser450 by mTORC2 [in addition to phosphorylation at Thr308 by PDK1 (Fig. 1)]. In order to be able to investigate specifically, the potential relationship of *PIK3CA* wild type/mutation status and PIK3CA/PTEN expression on AKT phosphorylation expression, an antibody for AKT phosphorylated at Thr308 only was selected.

**Table 2:** Details of immunohistochemical procedures

Antigen	Antibody (clone)	Supplier	Antigen retrieval	Antibody dilution, incubation time
EGFR	Mouse mAb (H11)	Dako North America Inc., Carpinteria, CA, USA	0.1% (w/v) trypsin in 40 mM CaCl_2_/TBS pH 7.8, 20 min, 37°C	1:100, 30 min RT then 4°C overnight
PIK3CA	Rabbit pAb (LS-B5363)	LifeSpan Biosciences, Inc., Seattle, WA, USA	Citrate buffer pH 6.0^a^	1:150, 30 min, RT
PTEN	Rabbit mAb (138G6)	Cell Signaling Technology, Danvers, MA, USA	Citrate buffer pH 6.0^a^	1:100, 40 min, RT
Phospho-AKT (Thr308)	Mouse mAb (18F3.H11)	Rockland Immunochemicals, Inc., Gilbertsville, PA, USA	None	1:100, 1 h, RT
Phospho-mTOR (Ser2448)	Rabbit mAb (49F9)	Cell Signaling Technology, Danvers, MA, USA	Citrate buffer pH 6.0^a^	1:100, 40 min, RT
Phospho-p44/42 MAPK (Erk1/2) (Thr202/Tyr204)	Rabbit mAb (D13.14.4E)	Cell Signaling Technology, Danvers, MA, USA	Citrate buffer pH 6.0^a^	1:400, 1 h, RT

^a^Heat treatment: 105°C for 20 min (Decloaking Chamber, Biocare Medical, Concord, CA), then 20 min cool down.

TMA sections were deparaffinized thorough xylene and rehydrated through graded ethanol followed by antigen retrieval. Slides were then treated with 3% hydrogen peroxide in PBS/0.05% tween 20 buffer to block endogenous peroxidase activity. After this, a 15 min non-specific protein binding blocking step was performed (Protein Block, serum-free [Dako North America Inc., Carpinteria, CA, USA]). Primary antibody was then applied followed by secondary detection using a Dako EnVision+/HRP/DAB+kit and counterstaining with hematoxylin. IHC assays were performed according to supplier recommendations; optimization tests were also performed on breast, colon, or tonsil TMA specimens as well as the CSCC TMAs. Negative control assays were performed using IgG antibodies to *Aspergillus niger* glucose oxidase (Dako North America Inc.).

IHC staining patterns were reviewed (independently by MFE, SB with support from KC) naïve to mutation data and were scored with reference to the proportion of cells staining: 0 = Negative; 1 = Rare cell positive (<1%); 2 = Focally positive (1–25%); 3 = Variably positive (25–75%); 4 = Uniformly positive (>75%); and in terms of staining intensity: 0 = Negative; 1 = weakly positive; 2 = moderately positive; and 3 = strong positive. These scores were summed to give an Allred score (AS) ranging from 0 to 7 [28]. Because IHC data interpretation is unstandardized, can be controversial and is dependent on the scoring approach, IHC staining was also categorized into low-level (LL) (AS <4) and high-level (HL) (AS ≥4) staining groups [29]. This dichotomous grouping approach was taken given the limiting numbers of specimens in the study. Staining was judged among tumors relative to each other as matched specimens of normal cervical epithelium from each patient were not available for study.

### Statistical analysis

Mutation and IHC data were cross-compared and compared against age, tumor grade (well, moderate, poor), stage (T1 vs. ≥T2), size (depth of invasion ≤2 cm vs. >2 cm), lymph node status (negative or positive). Patient outcome data was inaccessible for the purposes of this study. Data were assessed by Fisher’s exact test, Mann–Whitney test, or Kruskall-Wallis test. Analyses were performed using InStat software (GraphPad Software Inc., La Jolla, CA, USA).

## Results

### Mutation analyses

*KRAS* mutations were detected in 0/91 (0%) informative specimens (i.e., that showed a Ct value ≤30 for the detection of *KRAS* in the absence of a PNA-Clamp oligo). *PIK3CA* mutations were detected in 19/95 (20.0%) informative specimens. *PIK3CA* mutations in exon 9 were the most common: 3 (15.8%) in E542 and 15 (78.9%) cases in E545; 1 (5.3%) specimen showed an exon 20 mutation at H1047R. Mutation status showed no correlation with FIGO stage, tumor size, differentiation or lymph node (LN) status. *PIK3CA* mutation detection was associated with older age: wild type mean age 48.2 (SD 13.7, age range 25–90) vs. mutation mean age 56.6 (SD 10.7, age range 45–80), P = 0.007. Dividing the patients as pre-menopausal vs. post-menopausal, mutations were detected in 5/45 (11.1%) women <50 years and in 14/50 (28%) ≥50 years (P = 0.045; OR = 3.111). *PIK3CA* mutation data in relation to clinical characteristics are summarized in Table 3 and Additional file 1: Table S1.

**Table 3:** Correlation between clinical characteristics and *PIK3CA* mutations

Characteristics	n = 95	*PIK3CA* mutant-type	*PIK3CA* wild-type	*P* value*
		No. of patient (n = 19)	No. of patient (n = 76)	
Mean age (SD)	49.7 (13.2)	56.6 (10.7)	48.2 (13.7)	*0.007*
FIGO stage				
Stage I	84 (88.4%)	16 (16.8%)	68 (71.6%)	0.688
Stage II–III	11 (11.6%)	3 (3.2%)	8 (8.4%)	
Tumor size				
≤2 cm	11	1	10	0.433
>2–5 cm	69	16	53	
>5 cm	15	2	13	
Lymphnode				
Positive	17	5	12	0.337
Negative	61	11	50	
No data	17			
Histology				
Well	16 (16.8%)	2 (2.1%)	14 (14.7%)	0.547
Moderately	65 (68.4%)	13 (13.7%)	52 (54.7%)	
Poorly	14 (14.7%)	4 (4.2%)	10 (10.5%)	

Italic value indicates statistically significant.

* Pearson chi square and Fisher’s exact test.

### Immunohistochemistry

Marker staining data in terms of ‘negative’, ‘low’, ‘intermediate’ and ‘high’ Allred scores is shown in Table 4.

**Table 4:** Summary of marker panel staining patterns

Marker	Negative	Low	Intermediate	High
EGFR (n = 101)	41 (40.6%)	22 (21.8%)	26 (25.7%)	12 (11.9%)
PIK3CA (n = 101)	1 (1.0%)	0 (0%)	13 (12.9%)	87 (86.1%)
PTEN (n = 100)	69 (69.0%)	5 (5.0%)	26 (26.0%)	0 (0%)
p-mTOR (n = 101)	37 (36.6%)	22 (21.8%)	41 (40.6%)	1 (1.0%)
p-AKT cytoplasmic (n = 101)	11 (10.9%)	5 (5.0%)	47 (46.5%)	38 (37.6%)
p-AKT nuclear (n = 101)	45 (44.6%)	4 (4.0)	31 (30.7%)	21 (20.8%)
p-MAPK/Erk1/2 cytoplasmic (n = 102)	31 (30.4%)	14 (13.7%)	57 (55.9%)	0 (0%)
p-MAPK/Erk1/2 nuclear (n = 102)	27 (26.5%)	11 (10.8%)	64 (62.7%)	0 (0%)

Each antibody was reviewed with respect to the proportion of cells staining: 0 = Negative; 1 = Rare cell positive (< 1%); 2 = Focally positive (1–25%); 3 = Variably positive (25–75%); 4 = Uniformly positive (>75%); and in terms of staining intensity: 0 = Negative; 1 = weakly positive; 2 = moderately positive; and 3 = strong positive. These scores were summed to give an Allred score (AS) ranging from 0 to 7. Staining data is summarized in the above table as: Negative, (AS = 0–1), Low (AS = 2–3), Intermediate (AS = 4–6), High (AS = 7).

### EGFR

EGFR stained positive (AS ≥1) in 60/101 (59.4%) specimens; 38/101 (37.6%) showed HL staining (AS ≥4); 55/60 (91.7%) tumors showed membranous and cytoplasmic staining, 2/60 (3.3%) showed cytoplasmic staining and 3/60 (5.0%) showed membranous staining. No significant correlations were detected with respect to patient age, tumor stage or size (p > 0.05).

EGFR staining showed a significant association with *PIK3CA* mutation status: wild type mean AS 2.4 (SD 2.6) vs. mutation mean AS 3.8 (SD2.7), P = 0.043. HL EGFR staining was noted in 12/19 (63.2%) *PIK3CA* mutated specimens and in 23/72 (31.9%) wild type specimens (P = 0.018; OR = 3.652).

EGFR staining showed an association with lymph node status: mean AS for LN negative patients was 2.9 (SD 2.5) vs. 1.7 (SD 2.5) for lymph node positive specimens (P = 0.047). On the basis of LL/HL categorization, 4/21 (19.0%) LN positive patients showed staining vs. 26/65 (40.0%) LN negative patients (P = 0.114; OR = 2.833).

**Fig. 2:**
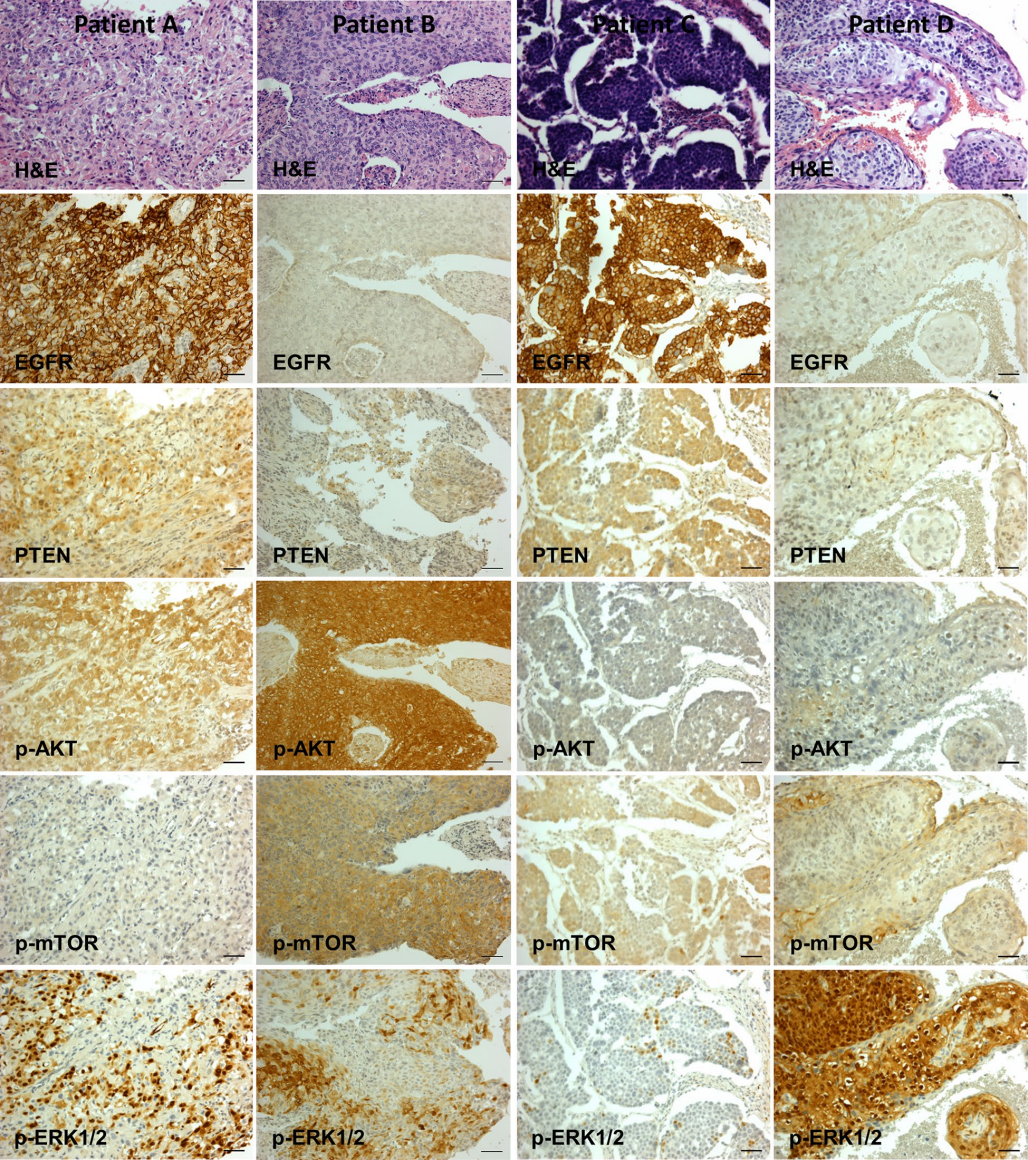
Representative histochemical and immunohistochemical staining. Heterogeneous staining pattern combinations were detected among 105 cervical squamous cell carcinomas. *Patients A* and *B*: moderately differentiated Stage 1 tumors, wild type *PIK3CA*; *patients C* (poorly differentiated) and *D* (moderately differentiated): Stage 1 tumor, mutated *PIK3CA.* *Scale bar* 50 µm. All images were originally photographed with a ×20 objective lens using an Olympus BX50 light microscope (Center Valley, PA, USA) equipped with a QImaging Retiga 2000R digital camera (Surrey, BC, Canada) (PIK3CA staining is included in Additional File 2: Figure S1).

AS data analysis showed an association of EGFR staining with histological grade: Well 4.3 (SD 2.5), Moderate 2.2 (SD 2.5), and Poor 3.4 (SD 2.8), P = 0.029; no significant trend was shown when the data were scored according to LL/HL, P = 0.351. Representative staining is shown in Fig. 2. Table 5 summarizes marker association data.

**Table 5:** Summary of staining patterns that included statistically significant associations

Marker	*PIK3CA* wild type (n = 19)	*PIK3CA* mutant (n = 72)		P value
EGFR mean AS	2.4 (2.6)	3.8 (2.7)		0.043*
EGFR HL AS	12	23		*0.018* ** (OR = 3.652)
EGFR LL AS	49	7		

	Lymph node negative (n = 65)	Lymph node positive (n = 21)		
EGFR mean AS	2.9 (2.5)	1.7 (2.5)		*0.047*
EGFR HL AS	26	4		0.114*
EGFR LL AS	39	17		

	Well differentiated (n = 8)	Moderately differentiated (n = 67)	Poorly differentiated (n = 26)	
EGFR mean AS	4.3 (2.5)	2.2 (2.5)	3.4 (2.8)	*0.029* ***
EGFR HL AS	5	21	12	0.351^a^,*
EGFR LL AS	3	46	14	

	Lymph node negative (n = 65)	Lymph node positive (n = 21)		
p-AKT cytoplasmic mean AS	4.9 (2.3)	5.8 (2.2)		*0.027* *
p-AKT cytoplasmic				
HL AS	52	18		0.751**
LL AS	13	3		

	Well differentiated (n = 8)	Moderately differentiated (n = 65/66)	Poorly differentiated (n = 27)	
p-AKT cytoplasmic mean AS	6.6 (0.5)	5.4 (2.0)	4.5 (2.3)	*0.009* ***
p-AKT cytoplasmic				0.518^a,^*
HL AS	8	56	22	
LL AS	0	9	5	
p-AKT nuclear mean AS	5.1 (3.1)	3.3 (3.0)	2.0 (2.7)	*0.014* ***
p-AKT nuclear				0.178^a,^*
HL AS	5	35	10	
LL AS	3	31	17	

AS (Allred Score), High Level (HL) AS (AS ≥4), Low Level (LL) AS (AS <4).

Italic values indicate statistically significant.

* Mann–Whitney test.

** Fisher’s Exact test.

** Kruskall-Wallis test.

^a^Well and moderate categories combined.

### Phospho-AKT

Cytoplasmic staining (AS ≥1) was detected in 90/101 (89.1%) CSSCs; 85/101 (84.2%) showed HL staining. No significant correlations were detected with respect to *PIK3CA* mutation status, patient age, tumor stage or size (p > 0.05). pAKT cytoplasmic staining showed an association with lymph node status: mean AS for LN negative patients was 4.9 (SD 2.3) vs. 5.8 (SD 2.2) for lymph node positive specimens; P = 0.027. On the basis of LL/HL categorization, 3/21 (14.3%) LN positive patients showed staining vs. 13/65 (20.0%) LN negative patients; P = 0.75. Cytoplasmic AS data analysis showed an association with histological grade: Well 6.6 (SD 0.5), Moderate 5.4 (SD 2.0), and Poor 4.5 (SD 2.3), P = 0.009; no significant trend was shown when the data were scored according to LL/HL, P = 0.518.

Nuclear pAKT staining (AS ≥1) was detected in 56/101 (55.4%); 50/101 (49.5%) showed HL staining. No significant correlations were detected with respect to PIK3CA mutation status, patient age, tumor stage, size or lymph node status (p > 0.05). Nuclear AS data analysis showed an association with histological grade: Well 5.1 (SD 3.1), Moderate 3.3 (SD 3.0), and Poor 2.0 (SD 2.7), P = 0.014; no significant trend was shown when the data were scored according to LL/HL, P = 0.085. Representative staining is shown in Fig. 2.

### PIK3CA, PTEN, phospho-mTOR, phospho-p44/42 MAPK (ERK1/2)

PIK3CA, PTEN, and p-mTOR, p-MAPK (ERK1/2) showed no significant relationships to mutation status or to any clinical parameter (P > 0.05). PIK3CA showed strong cytoplasmic staining (AS ≥4) in 100/101 (99.0%) specimens (Additional file 2: Figure S1). PTEN stained (mostly cytoplasmic, occasional nuclear) positive (AS ≥1) in 31/100 (31.0%) specimens; 26% showed HL staining. mTOR stained (mostly cytoplasmic, occasional nuclear) positive (AS ≥1) in 64/101 (63.4%) specimens; 42/103 (40.8%) showed HL staining. P-MAPK cytoplasmic staining was detected in 71/102 (69.6%) and 59/102 (57.8%) CSSCs (AS ≥1 and AS ≥4 respectively.) P-ERK1/2 nuclear staining was detected in 75/102 (73.5%) and 64/102 (62.7%) CSSCs (AS ≥1 and AS ≥4 respectively). Representative staining is shown in Fig. 2.

### Inter-relationships of IHC markers

EGFR over-expression may activate the PI3K/AKT/mTOR pathway. No significant relationships were noted with respect to EGFR staining and PIK3CA, p-AKT or p-mTOR. Neither was PIK3CA staining related to p-AKT or p-mTOR, nor p-AKT to p-mTOR (P < 0.05). Furthermore, EGFR staining levels showed no correlation with pMAPK staining and there was also no significant association of PTEN expression with PIK3CA or pAKT.

## Discussion

This study has investigated CSCC specimens for aberrations to key EGFR pathway elements. EGFR IHC staining patterns were examined in relation to *KRAS* mutations and MAPK pathway expression, as well as in relation to *PIK3CA* mutations and PIK3CA, PTEN, pAKT and p-mTOR expression.

Low-level (LL) EGFR staining was present in 59.4% and high-level (HL) staining in 37.6% of the CSCC specimens. On the basis of mean Allred scores, EGFR staining was associated with well differentiated tumors and with lymph node negative patients. Previous EGFR IHC studies of cervical carcinomas show a wide range of expression levels (6–100%) [16, 18]; these data may be a reflection of differences in antibody clone choices, IHC protocols, and/or in how staining was rated [16]. HPV genotype may also be important as HPV16 E5 or E6/E7 proteins can lead to EGFR overexpression [20, 21]; our samples were all HPV16 positive, whereas other studies did not report or included a variety HPV types. There are also contradictory data about the relationship of EGFR expression to clinical markers, prognosis and therapeutic response: many studies show that EGFR overexpression is an independent predictor of poor response to radio/chemo-therapy, poor disease-free survival and poor overall survival; other studies show do not show these correlations [16]. Our data are supportive that EGFR staining could be used to identify CSCC patients eligible for anti-EGFR therapies.

The potential of anti-EGFR therapies for CSCC treatment is strengthened by the non-detection of *KRAS* mutations among our specimens. This finding is consistent with previous studies (Table 6) that have found *KRAS* mutations to be absent or uncommon (1.3–13.6%) in CSCC; *KRAS* mutations may occur more frequently in cervical adenocarcinomas (Table 6). *KRAS* mutations can chronically activate KRAS expression [9]. Via B-Raf, this in turn leads to continuously activated MAP kinase signaling as a consequence of phosphorylation in the cytoplasm of MEK1/2 and thence ERK1/2 and which in turn results in the activation of transcription factors promoting cell proliferation following migration into the cell nucleus [30]. In the absence of *KRAS* mutations it might therefore be expected that over-expression of EGFR would correlate with the expression of phosphorylated ERK1/2; this type of invariable simple linear relationship was not detected (Fig. 2). However, approximately 60% (HL) to 75% (LL) CSCCs stained negatively (cytoplasm or nucleus) suggesting the possibility that ERK1/2 staining might be used for identifying patients likely to benefit from anti-MAPK/ERK interventions. We are unaware of any other studies to date that have assessed pERK staining in cervical cancer.

**Table 6:** *KRAS* and *PIK3CA* mutations in cervical cancer

References	Squamous cell carcinoma		Adenocarcinoma/other	
	*KRAS* mutation	*PIK3CA* mutation	*KRAS* mutation	*PIK3CA* mutation
Dokianakis et al. [50]^a^	0/10 (0%)^1^	–	2/2 (100%)^1^	–
Stenzel et al. [51]^b^	3/22 (13.6%)^2^	–	1/2 (50.0%)^2^	–
Pappa et al. [52]^a^	2/28 (7.1%)^3^	–	1/19 (5.3%)^3^	–
Miyake et al. [53]^c^	–	2/12 (16.7%)^i^	–	1/9 (11.1%)^i^
Cui et al. [38]^d^	–	5/84 (6.0%)^ii^	–	10/100 (10.0%)^ii^
Iida et al. [18]^c^	0/32 (0%)^4^	–	3/48 (6.3%)^4^	–
Janku et al. [54]^e^	–	^i^2/8 (25.0%)	–	0/7 (0%)^i^
Janku et al. [55]^e^	1/10 (10%)^4^	5/14 (35.7%)^i^	–	–
Janku et al. [40]^e^	–	6/18 (33.3%)^i^	–	–
Wright et al. [19]^e^	0/40 (0%)^5^	15/40 (37.5%)^iii^	7/40 (17.5%)^5^	10/40 (25.0%)^iii^
McIntyre et al. [39]^f^	–	16/69 (23.2%)^i^	–	3/13 (23.1%)^i^
Ojesina et al. [48]^g^	1/79 (1.3%)^6^	9/79 (11.4%)^iv^	2/35 (5.7%)^6^	8/35 (22.9%)^iv^
Rashmi et al. [32]^e^	–	6/120 (5.0%)^iii^	–	2/20 (10.0%)^iii^
Spaans et al. [47]^h^	9/205^ns^ (4.4%)^5^	50/205^ns^ (24.4%)^iii^	Ns	Ns
Tornesello et al. [56]^i^	–	3/55 (5.5%)^v^	–	–
The present study^j^	0/91 (0%)^7^	19/95 (20.0%)^vi^	–	–

Specimen country of origin: ^a^ Greece, ^b^ Poland, ^c^ Japan, ^d^ Sweden, ^e^ USA, ^f^ Canada, ^g^ Norway and Mexico, ^h^ The Netherlands, ^i^ Italy, and ^j^ India. *KRAS* mutation methodology: ^1^ codon 12 PCR/sequencing, ^2^ codon 12 PCR–RFLP/SSCP, ^3^ codons 12, 13 and 16 PCR/sequencing, ^4^ codons 12 and 13 PCR/sequencing, ^5^ sequenom MALDI-TOFF massARRAY, ^6^ exome/whole genome Illumina HiSeq 200, ^7^ codons 12 and 13 PNA-Clamp PCR. *PIK3CA* mutation methodology: ^i^ exons 9 and 20 PCR/sequencing, ^ii^ exons 1, 9 and 20 PCR/sequencing, ^iii^ sequenom MALDI-TOF massARRAY, ^iv^ exome/whole genome Illumina HiSeq 200, ^v^ exon 9 nested PCR/sequencing, ^vi^ exons 9 and 20 PNA-Clamp PCR. ^ns^ not specified whether specimens were squamous cell carcinoma or adenocarcinoma.

PIK3CA mutations were detected in 20.0% of 95 CSCC specimens. As with IHC, mutation data estimates may be impacted by the experimental technique. Most studies of *KRAS* and *PIK3CA* mutations in cervical cancer have combined PCR amplification across mutation hotspot regions with Sanger sequencing (Table 6). The potential limitation of this approach is the detection sensitivity, which is of the order ~10% [31]. The highest and lowest estimates of *PIK3CA* mutation frequencies in cervical cancers (5.7 and 37.5%) have been obtained using Sequenom massARRAY technology [19, 32], which has a sensitivity threshold of ~5% [33]. The detection sensitivity for methods employing massively parallel sequencing is of the order ~1% [31], which is the same as the analytical sensitivity reported for the PNA-Clamp technique [34]. *PIK3CA* mutations may activate the PI3K-PTEN-AKT pathway; however, there are contradictory data as to whether or not *PIK3CA* mutations confer anti-EGFR therapy resistance [35–37].

The *PIK3CA* and *KRAS* mutation detection rates among the Indian CSCCs is comparable to what has been found in MDC CSCCs (Table 6) suggesting that susceptibility to the accumulation of these mutations in CSCC aetiology is an intrinsic biologic property of the disease rather than related to ethnicity or culture. *PIK3CA* mutation detection in our study was associated with older, post-menopausal age. The possibility of a relationship to older age has previously been suggested on the basis of data combined from the detection of *PIK3CA* mutations in 5/84 (6.0%) CSCC and 10/100 (10.0%) cervical adenocarcinomas in patients from Sweden [38] suggesting age may be a global determinant for *PIK3CA* mutation susceptibility in cervical cancer. These data indicate that *PIK3CA* mutation prevalence estimates may be ‘biased’ by the age of the sample population. Among our specimen set, *PIK3CA* mutations did not correlate with any available clinical parameters. In previous studies, *PIK3CA* mutations have been found associated with shorter survival [19], or with better survival or treatment response for patients treated with radical chemoradiotherapy or PI3K/AKT/mTOR inhibitors [39–41]. These findings together with the discovery of mutations in 20% of CSCCs are supportive for the use of *PIK3CA* mutation testing for prognostic stratification and as a potential target for therapeutic interventions. *PIK3CA* amplification has been reported in 14/20 (70.0%) [42] CSCCs; however, by IHC PIK3CA staining was strong in 99.0% CSCCs specimen in our study.

In relation to the EGFR/AKT/mTOR signaling pathway, *PIK3CA* mutations were associated with EGFR positive staining assessed either in in terms of AS (P = 0.043) or by LL vs. HL values (P = 0.018; OR = 3.652). The finding of a significant association with either form of IHC rating, strengthens support for a relationship between EGFR expression and *PIK3CA* mutation acquisition. Possibly, continuous PI3K synthesis together with increased cell proliferation heightens susceptibility to a *PIK3CA* mutation event. These data are also suggestive that patients with EGFR overexpression and *PIK3CA* mutations might usefully be treated synergistically using drugs for both targets.

PTEN staining was detected in a minority of specimens and was not associated with any of the clinical parameters. Absence of expression of this tumor suppressor protein that dephosphorylates PIP3 preventing AKT activation is consistent with tumor promotion. Other studies have found low PTEN expression in CSCC but have also noted an association of expression with tumor size and lymph node involvement [43, 44]. Loss of heterozygosity at the *PTEN* locus has been shown up to 23% of Indian cervical cancers [45] and *PTEN* promoter methylation in 58% of CSCC [46]. *PTEN* mutations in cervical cancer have been reported in 2.4–6.9% of specimens [47, 48]. Accordingly, PTEN IHC biomarker data might also usefully be figured into a drug selection for CSCC.

Elevated EGFR, in the absence or presence of PTEN expression, did not correlate with elevated phospho-AKT and phospho-mTOR expression. Cytoplasmic pAKT expression was common and was associated with lymph node positive patients (P = 0.022) and with well differentiated tumors when data was assessed in terms of AS values but no such association was found when the data was assessed by LL/HL cut-off points. Nuclear pAKT (detected in ~50% of the tumors) also showed an association with well differentiated tumors by AS assessment only (P = 0.046). P-mTOR showed staining in 40.8% (HL)–59.2% (LL) of the specimens and was unassociated with any study parameters.

In this study we focused on some of the factors that impact EGFR related pathways. Intercellular signaling pathways, such as the MAPK/ERK and PI3K/AKT/mTOR, are circuitously inter-related and impacted directly or indirectly by multiple other factors, genetic and epigenetic. Consequently, the expression of downstream events in a pathway may be the outcome of other over-riding (aberrant) parallel pathway processes or meta-levels of regulation rather than being directly related to an upstream amplification or mutation event. For example, in the case of cervical disease, the expression of other RTKs such as ErbB-2, or enzymes such as cyclooxygenase-2 may impact pathway expression [57]. Additionally, CSCC results from chronic infection with high-risk HPV. The effects of the HPV16 *E5*, *E6* and *E7* oncogenes have been well characterized for their dysregulation of EGFR, p53 and pRB respectively. The HPV oncogenes can also impact multiple additional cellular pathway factors [58, 59]. It is therefore unsurprising that CSCC shows manifold heterogeneity as reflected in EGFR pathway expression profiles.

In summary, the major findings of this study are threefold. Firstly, *KRAS* mutations were undetected in Indian CSSCs whereas *PIK3CA* mutations were age-associated and relatively common being detected in 28.0% of specimens from women ≥50 years vs. 11.1% of women <50 years. Secondly, across patients EGFR staining was common and was associated with *PIK3CA* mutation detection (by mean AS or by LL/HL scoring) and also showed associations with lymph node status and histological grade. pAKT IHC staining also showed association with lymph node status and histological grade. These relationships were partially dependent on how IHC staining patterns were scored reaffirming the requirement for standardized IHC protocols to allow accurate biomarker inter-study comparisons and clinical appraisal [49]. Thirdly, at the level of individual patients, qualitatively distinct marker panel staining profiles were observed patient to patient (Fig. 2). This finding may be of significance for precision/individualized/personalized medicine. A variety of cellular pathways are candidates for pharmacologic interventions and for each pathway there may be multiple potential actionable targets. Our data suggest that in addition to molecular profiling, IHC data might be usefully incorporated into precision medicine algorithms; the expression level of putative pathway targets may be important for deciding among therapeutic options. For example, Patient A (Fig. 2) might be a candidate for EGFR or ERK targeted therapies, Patient B for AKT, mTOR or ERK, Patient C for EGFR or mTOR, and Patient D for ERK therapies. Clinical trials in particular might benefit from noting pre- and post-treatment expression levels of intervention pathways/targets and the relationship to therapeutic response.

## Conclusions

This study shows that EGFR represents a promising target for CSCC treatments, especially given the absence of *KRAS* mutations among the study samples. It is unclear whether *PIK3CA* mutations compromise EGFR therapies. However, pharmacologic agents used to treat CSCC patients with *PIK3CA* mutations have been shown to be efficacious [41]; these together with EGFR treatments might therefore be usefully combined. Downstream cellular pathway protein expression levels may not necessarily be predictable on the basis of amplification events or activating mutations. IHC or proteomic assay of these expression levels may be of importance for selecting among alternative therapeutic strategies for use in precision medicine protocols. Overall, the data for the Indian CSCCs is comparable to data published for CSCCs from more developed countries suggesting that EGFR pathway-related therapies could be of benefit to patients worldwide.

## Additional files

Additional file 1:
**Table S1.** *KRAS* and *PIK3CA* mutations detected. Additional file 2:
**Figure S1.** Representative histochemical and immunohistochemical staining. Heterogeneous staining pattern combinations were detected among 105 cervical squamous cell carcinomas. Patients A and B: moderately differentiated Stage 1 tumors, wild type *PIK3CA*; patients C (poorly differentiated) and D (moderately differentiated): Stage 1 tumor, mutated *PIK3CA.* Scale bar: 50 µm. All images were originally photographed with a X20 objective lens using an Olympus BX50 light microscope Center Valley, PA) equipped with a QImaging Retiga 2000R digital camera (Surrey, BC).

## Abbreviations

HPV: human papillomaviruses
CSCC: cervical squamous cell carcinoma
MDCs: more developed countries
LDCs: less developed countries
FFPE: formalin-fixed, paraffin-embedded
PNA: peptide nucleic acid
TMA: tissue microarray
IHC: Immunohistochemistry
LN: lymph node status
AS: Allred Score
LL: low-level
HL: high-level
