# Supplementary material for: Epidermal growth factor receptor pathway mutation and expression profiles in cervical squamous cell carcinoma: therapeutic implications
Source: J Transl Med. 2015 Jul 25;13:244. doi: 10.1186/s12967-015-0611-0 (PMC4513684; doi:10.1186/s12967-015-0611-0)
Supplement: Additional file 1: — Table S1. KRAS and PIK3CA mutations detected. [file 12967_2015_611_MOESM1_ESM.docx]

**Additional file 1 Table S1** ***KRAS* and *PIK3CA* mutations detected**

| **Patient No.** | **Age (years)** | **Histology** | ***KRAS* mutation** | ***PIK3CA* mutation** |
| --- | --- | --- | --- | --- |
| 1 | 65 | Moderately Differentiated | None | E545 |
| 2 | 60 | Poorly Differentiated | None | E545 |
| 3 | 60 | Poorly Differentiated | None | E545 |
| 4 | 80 | Moderately Differentiated | None | H1047 |
| 5 | 40 | Moderately Differentiated | None | E545 |
| 6 | 45 | Well Differentiated | None | E545 |
| 7 | 50 | Moderately Differentiated | None | E545 |
| 8 | 65 | Moderately Differentiated | None | E545 |
| 9 | 50 | Moderately Differentiated | None | E545 |
| 10 | 55 | Poorly Differentiated | None | E545 |
| 11 | 65 | Moderately Differentiated | None | E542 |
| 12 | 42 | Well Differentiated | None | E545 |
| 13 | 60 | Moderately Differentiated | None | E545 |
| 14 | 60 | Poorly Differentiated | None | E545 |
| 15 | 45 | Moderately Differentiated | None | E542 |
| 16 | 70 | Moderately Differentiated | None | E545 |
| 17 | 45 | Moderately Differentiated | None | E542 |
| 18 | 65 | Moderately Differentiated | None | E545 |
| 19 | 54 | Moderately Differentiated | None | E545 |
