# Supplementary figures and images for: Epidermal growth factor receptor pathway mutation and expression profiles in cervical squamous cell carcinoma: therapeutic implications
Source: J Transl Med. 2015 Jul 25;13:244. doi: 10.1186/s12967-015-0611-0 (PMC4513684; doi:10.1186/s12967-015-0611-0)

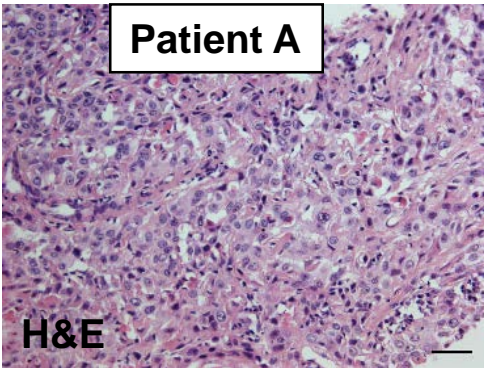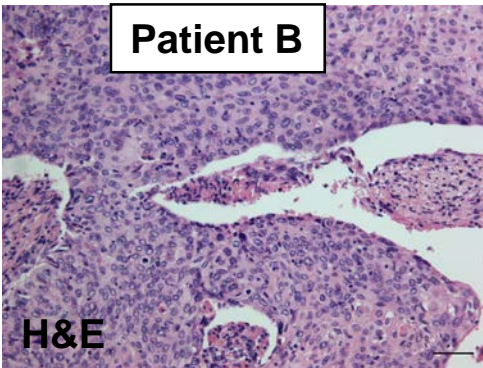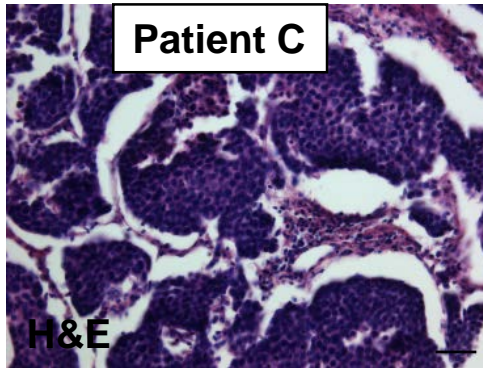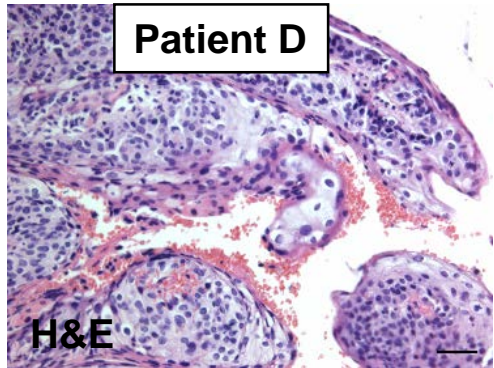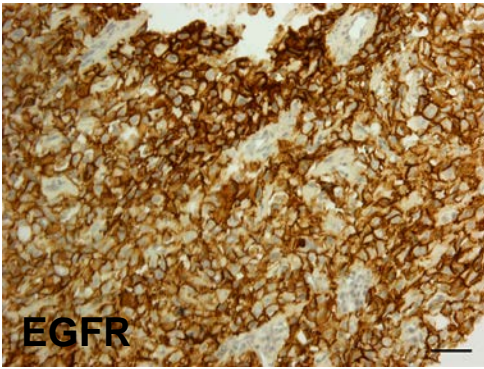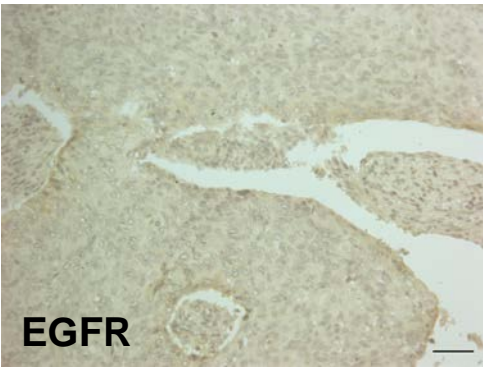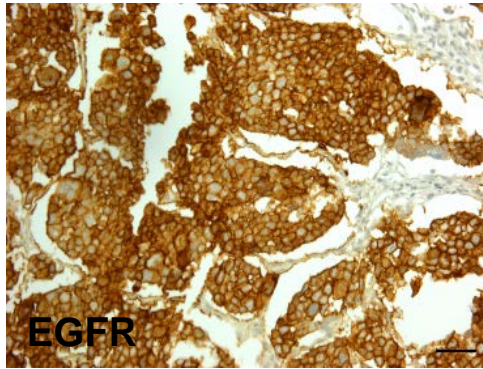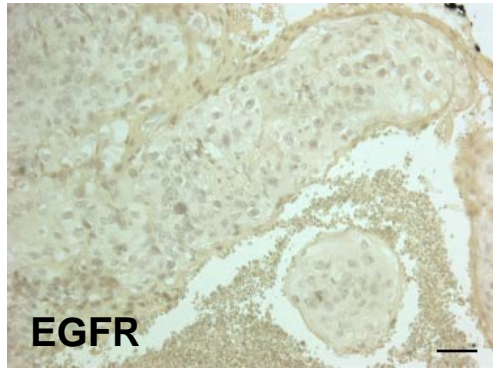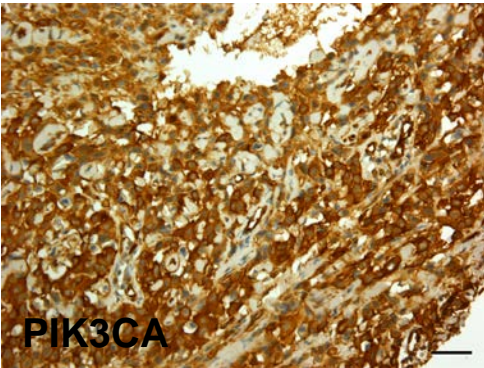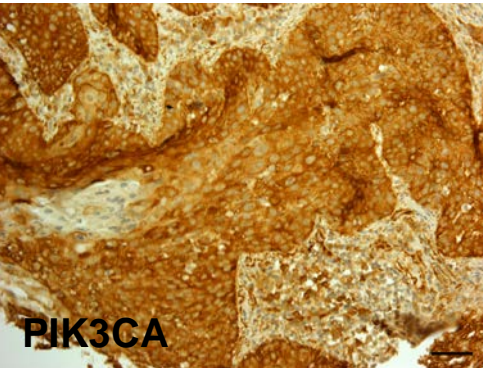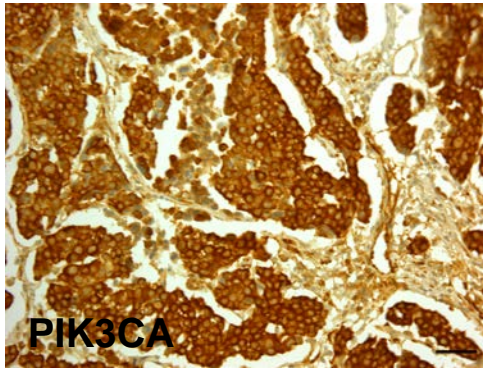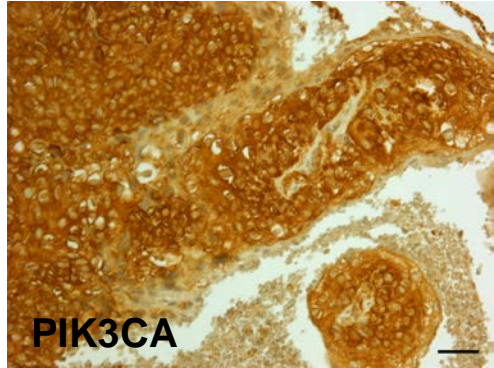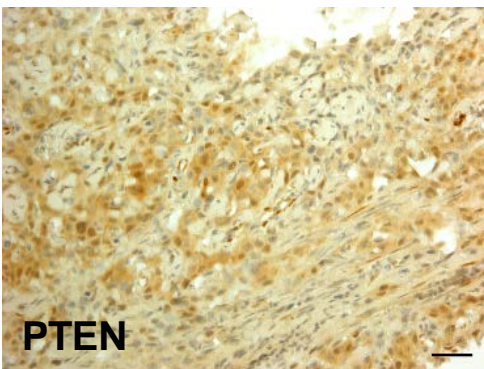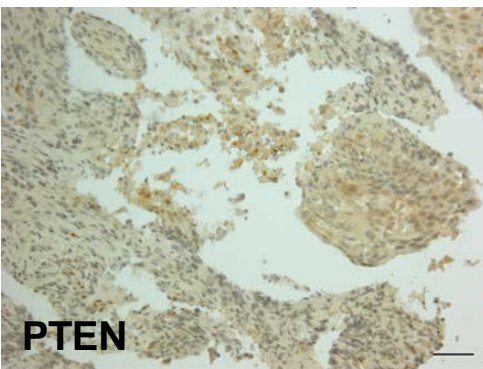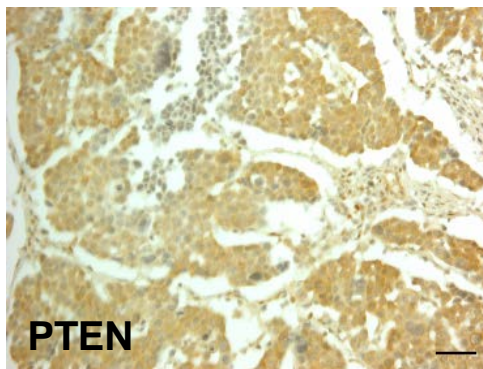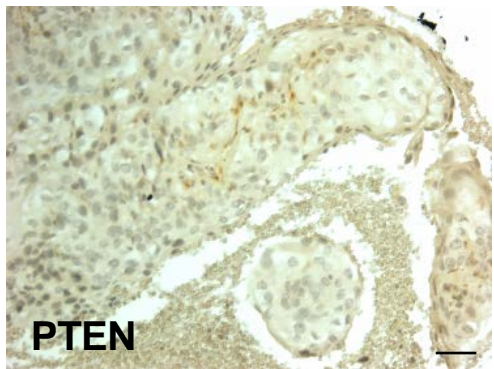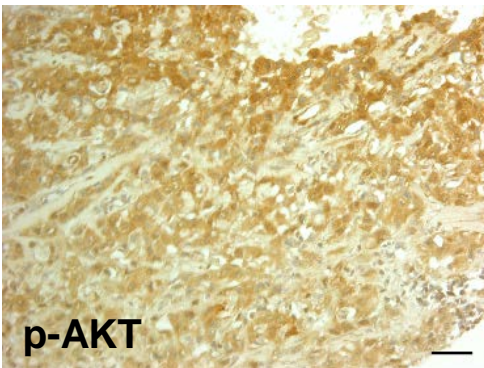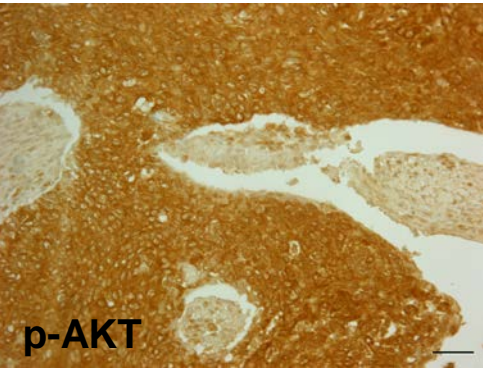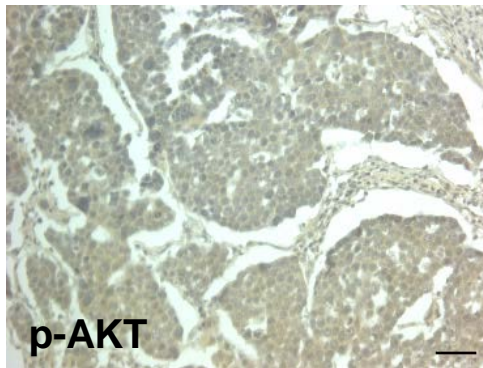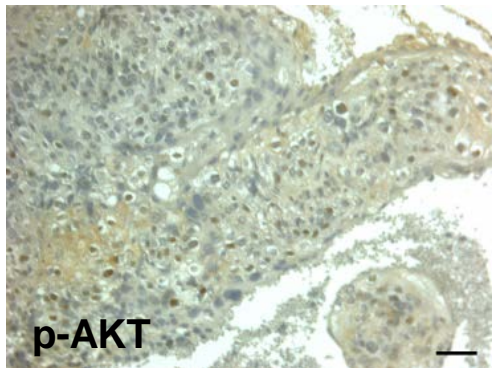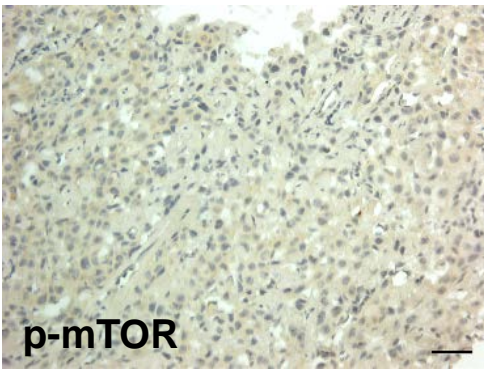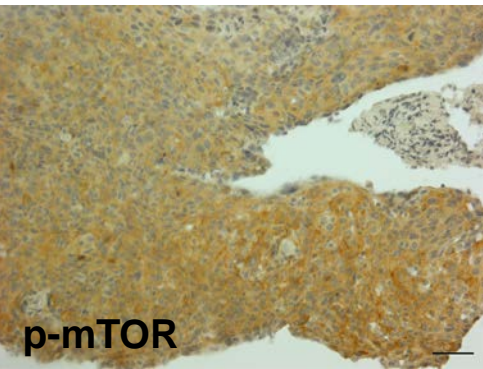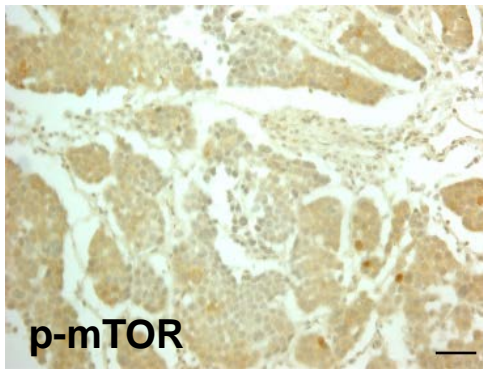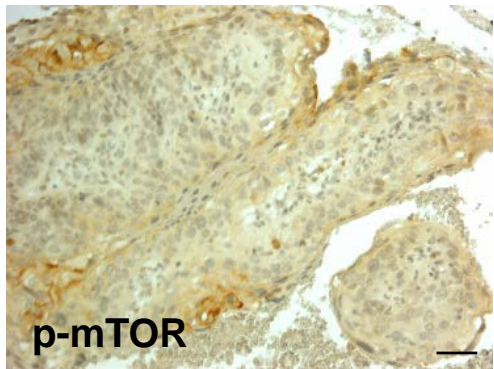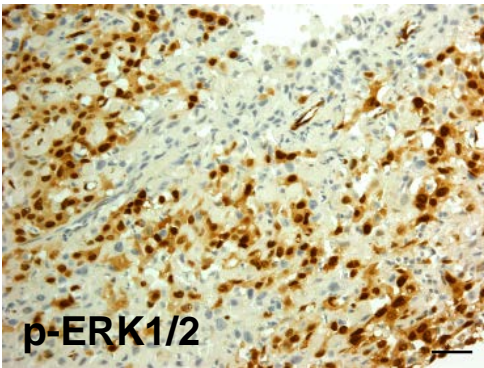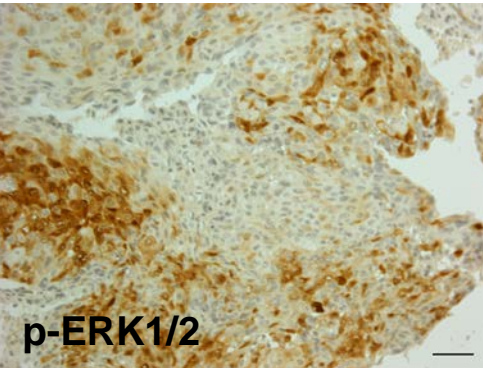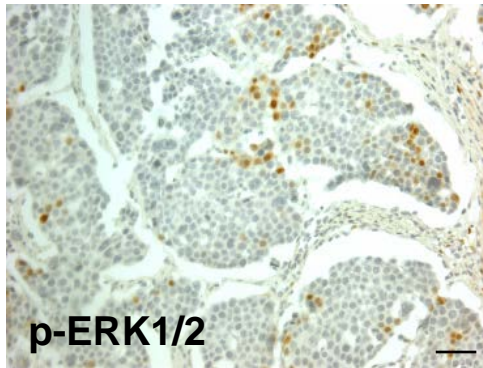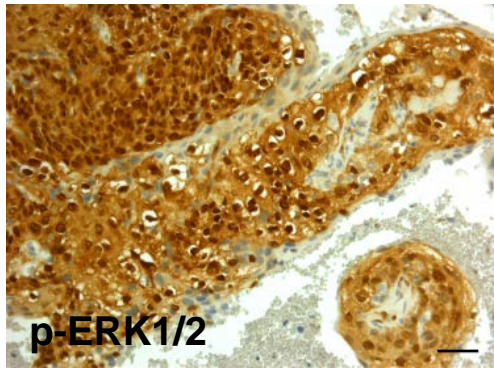

Supplement: Additional file 2: — Figure S1. Representative histochemical and immunohistochemical staining. Heterogeneous staining pattern combinations were detected among 105 cervical squamous cell carcinomas. Patients A and B: moderately differentiated Stage 1 tumors, wild type PIK3CA; patients C (poorly differentiated) and D (moderately differentiated): Stage 1 tumor, mutated PIK3CA. Scale bar: 50 µm. All images were originally photographed with a X20 objective lens using an Olympus BX50 light microscope Center Valley, PA) equipped with a QImaging Retiga 2000R digital camera (Surrey, BC). [file 12967_2015_611_MOESM2_ESM.pdf]
